# 18.3 DISTURBED AND INTACT ATTENTIONAL PROCESSES DURING WORKING MEMORY ENCODING IN SCHIZOPHRENIA: CONVERGING BEHAVIORAL AND IMAGING GENETICS EVIDENCE

**DOI:** 10.1093/schbul/sby014.072

**Published:** 2018-04-01

**Authors:** Robert Bittner, Peter Hahn, Catherine Barnes, Tom Lancaster, David Linden, Andreas Reif

**Affiliations:** 1 University Hospital Frankfurt, Goethe University; 2 MRC Centre for Neuropsychiatric Genetics & Genomics, Cardiff University School of Medicine

## Abstract

**Background:**

Working memory and attention are fundamental and closely linked cognitive domains. This close link is exemplified by the crucial role of selective attention for the selection of information to be encoded into working memory. Patients with schizophrenia are markedly impaired in many aspects of both domains. However, the interplay between these cognitive deficits on both the cognitive and neurophysiological level remains poorly understood. Based on our previous findings regarding the central role of impaired working memory encoding to working memory dysfunction in schizophrenia, we hypothesize, that impaired attentional processes contribute to working memory dysfunction specifically during the encoding stage. This hypothesis was tested in both a behavioral experiment and in an fMRI imaging genetics study.

**Methods:**

For the behavioral study, we investigated 35 patients with schizophrenia and 35 matched healthy controls. In a change detection task, participants were simultaneously presented with both highly salient and non-salient spatial information. They were instructed to encode either the highly salient or the non-salient information. In half of the conditions, they were aided by a cue pointing them towards the relevant information. Our goal was to test, whether patients with schizophrenia were biased toward a particular type of information and whether a top-down cue would influence such a bias. In the imaging genetics study, we investigated 100 right-handed individuals without personal or family history of psychiatric disorders, who performed a visuospatial change detection task. The fMRI data were preprocessed and analyzed using Brain Voyager QX 20. For genotyping we used a custom Illumina HumanCoreExome-24 BeadChip array. We calculated polygenic scores (PGS) for schizophrenia based on 108 loci associated with schizophrenia in a recent mega-analysis of the Psychiatric Genetic Consortium (PGC2). We computed whole brain correlations between BOLD activation and PGS to elucidate the relationship between genetic risk for schizophrenia and abnormal brain function.

**Results:**

In the behavioral study, patients were significantly more impaired when required to encode non-salient compared to salient information. However, this impairment was specific to conditions without a top-down cue. This demonstrates, that patients could use top-down attention to overcome a bottom-up bias towards highly salient information during working memory encoding. In the fMRI data, we observed a significant negative correlation between BOLD activation in the right temporo-parietal junction (TPJ) during working memory encoding and PGS for schizophrenia. Across all subjects, this area showed robust deactivation during encoding. The TPJ is a crucial region of the ventral attention network and is closely involved in bottom-up attentional processes. Previous fMRI studies observed stronger deactivation of the TPJ during working memory encoding with increasing cognitive demand. Therefore, our results indicate that participants with a higher genetic risk for schizophrenia had to commit more cognitive resources by downregulating their ventral attention network.

**Discussion:**

Taken together, the results of both studies point toward specific disturbance of bottom-up attention during working memory encoding, which might be linked to genetic risk for schizophrenia. Conversely, top-down attention appears to be relatively spared. These findings provide new constraints for cognitive and neurophysiological models of impaired working memory encoding in schizophrenia.

